# Determination of orthodontic bracket and tooth adaptation using an X-ray micro-computer tomography scanner

**DOI:** 10.1016/j.mex.2020.100851

**Published:** 2020-03-06

**Authors:** Yaseen Fakir, Colm Keanly, Riaan Mulder

**Affiliations:** a Department of Orthodontics, The University of the Western Cape, Cape Town, South Africa; b X-Sight X-ray Services, Cape Town, South Africa; c Department of Restorative Dentistry, The University of the Western Cape, Cape Town, South Africa

**Keywords:** Orthodontic bracket, Teeth, Mesial curvature, Gingival curvature, Digital image processing

## Abstract

The determination of the orthodontic brackets’ adaptation to the curvatures of teeth is a difficult topic to study. The complexity arise due to the different designs of their fitting surfaces, margins and curvatures of orthodontic brackets. Teeth on the other hand have variation in their curvatures and the question remains how well an orthodontic brackets truly adapt to the teeth. Previous methods from the literature determined the curvature of teeth through the superimposition of circular templates onto dental plaster models as well as the use of acrylic arcs of various diameters. Here the authors describe a method to compare the curvatures obtained from an industrial X-ray micro-computer tomography scanner and processing software of orthodontic brackets and plaster casts of teeth. Three orthodontic brackets could be assessed by establishing co-ordinate points of proposed standardized landmarks on the orthodontic brackets. These standardized landmarks from each orthodontic bracket can be applied onto the various plaster teeth that was scanned. The adaptation of the three brackets to the various teeth can be compared by looking at their radii and angles that were determined in this method.

• The analysis of orthodontic brackets and teeth curvatures have been streamlined on a virtual platform.
• Analysis of X-ray micro-computer tomography of tooth curvatures provide a guide to the range of curvatures that orthodontic brackets need to fit.
• X-ray micro-computer tomography of orthodontic brackets can enhance the design of the brackets to fit a large array of tooth curvatures.

## Graphical abstract

**Figure:**
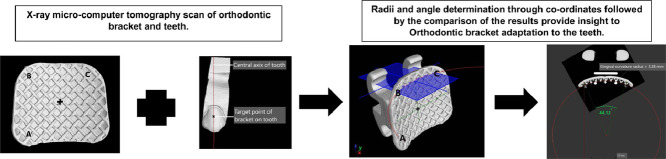

**Table:** Specifications Table

Subject area	*Medicine and dentistry*
More specific subject area:	*Dentistry - Orthodontics*
Method name:	*Orthodontic bracket radii and angle determination*
Name and reference of original method:	*du Plessis, A., Broeckhoven, C. & le Roux, S. G. Snake fangs: 3D morphological and mechanical analysis by microCT, simulation, and physical compression testing. Eur. J. Oral Sci. 7, 499–511 (2001)* *Watanabe, K. & Koga, M. A Morphometric Study with Setup Models for Bracket Design. Angle Orthod. 71, 499–511 (2001).*
Resource availability:	*Industrial X-ray micro-computer tomography scanner.* *Appropriate software program for analysis of scan data.*

## Method details

The industrial X-ray micro-computer tomography is a well-known method for assessment of various dental products and has been used successfully with dental materials for void determination [1,2]. It has become a widely accepted tool to investigate internal and external structures in the field of metrology [3]. The accuracy of the industrial X-ray micro-computer tomography has been shown to have improved quality of scans over medical CT scans [4]. Previous methods used to determine the curvature of teeth included the superimposition of circular templates onto dental plaster models as well as the use of acrylic arcs of various diameters [5]. The “best-fit” circle has been applied previously in the Volume graphics VG Studio software to measure the length of snake fangs with a cross-sectional sliced image from the scan [6]. However, the “best-fit” circle has never been used to determine the curvature of the orthodontic brackets’ fitting surface nor their adaptation to teeth from the scans obtained from X-ray micro-computer tomography. Here, we provide a sequential workflow procedure to determine a suggested standardized bracket dimension area, which eliminates the varied orthodontic bracket designs found between manufacturers. The method additionally allow comparison of the adaptation analysis of any orthodontic bracket to various teeth. The clinical implication of bracket design stems from research depicting a direct relationship between the curvature of the bracket, adaptation of the bracket to the surface of teeth and the resistance to the application of forces [7]. The method used is important to orthodontic bracket design, since no preceding studies can be found that relates the curvatures of commercially available orthodontic brackets to that of their corresponding teeth. The importance of this method is the interplay relationship of the orthodontic bracket base to the curvature of the corresponding area on the tooth. This interplay is of vital importance as similar curvatures of the bracket and tooth will lead to better adaptation of the bracket on the tooth. This has been established with the improved adaptation, increasing the sheer bond strength, which lead to an increased ability to endure orthodontic and masticatory forces from custom made orthodontic brackets as compared to conventional orthodontic brackets [8,9]. This agreed to the findings of another study investigating the shear bond strength of custom manufactured brackets versus conventional brackets and found less debonding in the custom bracket group. The study found an inverse relation between the adhesive thickness and sheer bond strength. The sheer bond strength increased as adhesive thickness decreased from 0.99 mm to 0.83 mm. However, this changed to a reduction in sheer bond strength when thickness of the adhesive was below 0.83 mm [10]. These studies highlight the importance of proper adaptation of the orthodontic bracket on the tooth in order to prevent excessive adhesive between the tooth and the bracket, hence no adhesive used for this method development.

## Required equipment and software

- Orthodontic brackets
- Plaster/gypsum / previously removed teeth
- Industrial or medical X-ray micro-computer tomography scanner
- Processing software for the analysis, in this method Volume graphics VG Studio max 3.2.5 (Heidelberg, Germany 2018).

The method here is described for the use with an industrial Nikon Metrology XTH 225 ST X-ray micro-computer tomography scanner (Yokohama, Japan) and Volume graphics VG Studio max 3.2.5, but any X-ray micro-computer tomography scanner and software for analysis can be used for image acquisition and analysis.

## Image acquisition

### Image acquisition of the teeth and brackets

1. For this study the appropriate tooth for the selected orthodontic bracket is the maxillary second premolar.
2. The model must be trimmed away until only the selected tooth remain, to reduce the scan time, as this comparison of bracket and tooth curvatures required the maxillary second premolar.
3. Each tooth is inscribed with a number for identification purposes, since there are models randomly selected to allow a varied sample of maxillary second premolar teeth.
4. The teeth are mounted on a firm foam holder in batches of three and scanned in the X-ray micro-computer tomography scanner (Fig. 1).

**Fig. 1:**
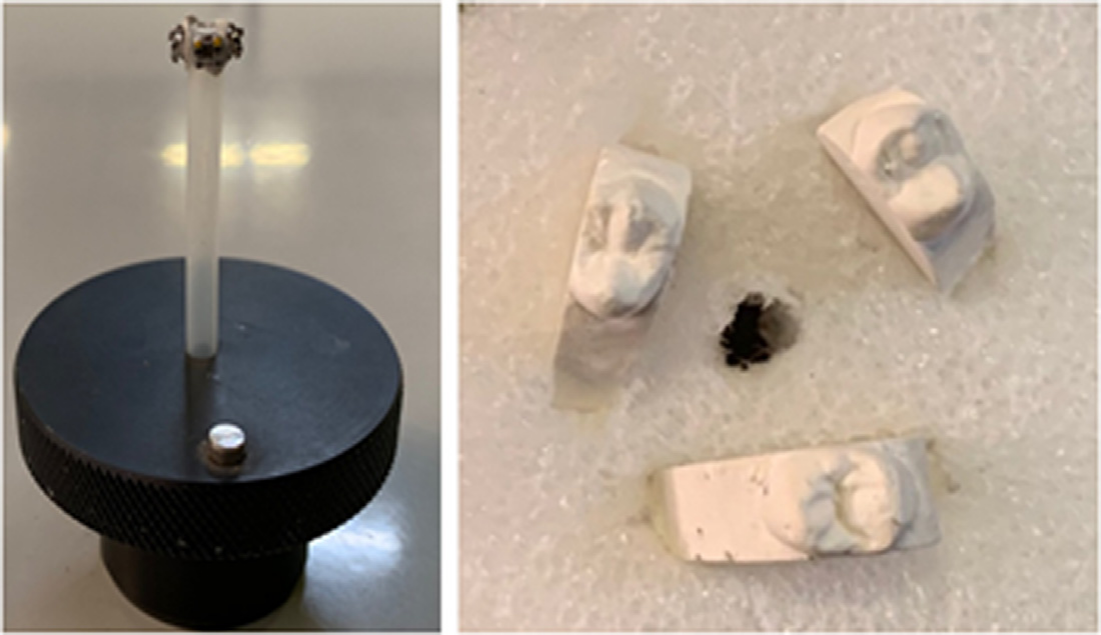
Teeth mounted on a firm foam holder in batches of three.
5. The X-ray micro-computer tomography scan duration for each teeth and bracket is 50 min with 2984 projections. To minimize scan time no detector shift is used. Copper filtration of 0.25 mm and 1 mm are used for the teeth and brackets respectively. The scans are taken at 100 kV with a beam current of 200µA and an exposure of 1fps. The voxel size for the tooth and bracket scans are 20 and 4.99 µm respectively with no beam hardening corrections necessary on the teeth scans.
6. With regard to the processing the signal noise ratio lends itself to avoid filtration, since the reconstructed volume consisted of only two peaks. The VG Studio (Volume graphics VG Studio max 3.2.5) selection modes are used to crop out the contour of the teeth and brackets and export the regions as a raw volume. This technique avoids “segmentation” as the internal structure of the teeth and brackets was not required for this study of bracket to tooth adaptation. The scan data is transferred to the software analysis program.
5. The teeth and brackets are scanned three and four at a time respectively and then separated, followed by individual analysis in the software by the advanced surface determination. This advanced surface determination feature is part of the co-ordinate measurement module, which is a licensed add-on module for geometry analysis. The specific tool used to measure the curvature of the surface of the bracket and teeth is the circle tool, which is also part of the co-ordinate measurement module.

## Measurement parameters of orthodontic brackets

### Height–width determination of orthodontic brackets

1. The height and width of each bracket is measured in the analysis software and recorded.
2. The height–width of the three orthodontic brackets are: Bioquick (Forestadent) 3.07 mm height/3.45 mm width; Innovation (GAC) 3.25/4.46 mm and Victory series (3 M Unitek) 2.88/3.29 mm

### Determination of the target point of the bracket and the standardized area of contact

Orthodontic brackets from different manufacturers have varied designs with regard to their respective shapes and edges. The area of measurement being investigated is chosen to be at 75% of the bracket width and height i.e. “standardized area of contact”. This is done in order to eliminate the influence of the variations in marginal designs of different brackets and would allow for measurement of an area of the bracket that should be well adapted to the surface tooth. However the “standardized area of contact” (area of measurement) can vary depending on the operator choice.

The standardized area of contact chosen is represented in this method by 75% of the height–width measurements and calculated as follows:

1. The “target point of the bracket” is the centre point of the bracket and the co-ordinates of this point and can be determined with the use of the height and width of the bracket, measured in the height–width determination step (Fig. 1).
2. The centre point of the bracket is located at the intersection of half the width and half the bracket height (Fig. 2). At this intersection a landmark point is placed.

**Fig. 2:**
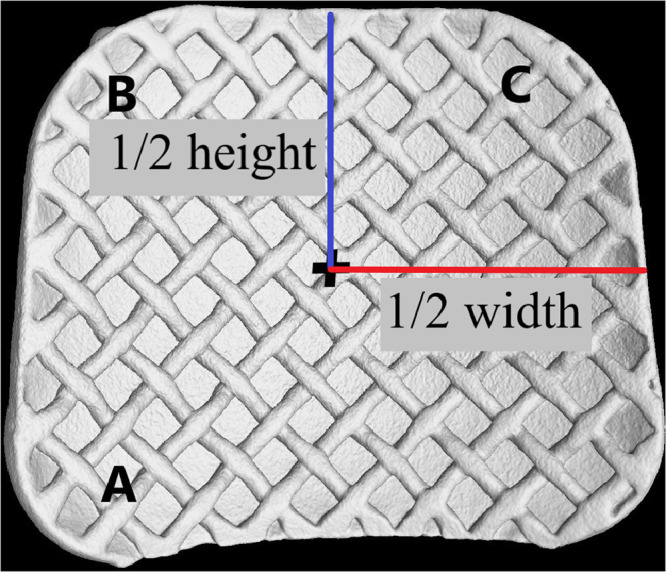
The centre point of the bracket is located.
3. The x, y and z coordinates of this landmark point, “the target point of the bracket”, is then recorded.
4. Height from target point of orthodontic bracket = 0.75 (height of orthodontic bracket / 2) represent the vertical offset of the bracket.
5. Width from target point of orthodontic bracket =0.75 (width of orthodontic bracket /2) represents the horizontal offset of the bracket.
6. The vertical and horizontal offsets represented by 75% of the height and width measurements are calculated for the three orthodontic brackets as represented in Table 1.

**Table 1:** Standardized area for the three orthodontic brackets.

Bracket	Height	Width
Victory series (3 M Unitek)	1.08 mm	1.23 mm
	(Vertical offset bracket: Victory series)	(Horizontal offset bracket: Victory series)
Innovation (GAC)	1.21 mm	1.30 mm
	(Vertical offset bracket: Innovation)	(Horizontal offset bracket: Innovation)
Bioquick (Forestadent)	1.15 mm	1.30 mm
	(Vertical offset bracket: Bioquick)	(Horizontal offset bracket: Bioquick)

### Determination of landmark points

1. Using the co-ordinates of the target point as the main reference point and the vertical and horizontal offsets for each bracket determined from the “75% of the bracket height and width”, the co-ordinates for three additional landmark points can be determined and placed on the bracket: Point A as the “inferior mesial margin”; Point B as the “gingival mesial margin” and Point C as the “gingival distal margin” on the orthodontic bracket (Fig. 2).
2. Table 2 identifies the co-ordinate calculation of the three additional landmarks (Points A, B and C) for the Victory series bracket.

**Table 2:** Co-ordinate calculation of the three additional landmarks for the Victory series bracket.

Point	Co-ordinate determination for Victory series bracket
Point A as the Inferior mesial margin	• X coordinate Victory series bracket = *X* of target +1.23mm(Horizontal offset Victory series bracket) • Y coordinate of Bracket *A* = *Y* of target −1.08mm(Vertical offset Victory series bracket) (Fig. 5).
Point B as the Gingival mesial margin	• X coordinate Victory series bracket = *X* of target +1.23mm(Horizontal offset Bracket A) • Y coordinate of Bracket *A* = *Y* of target +1.08 mm (Vertical offset Victory series bracket) (Fig. 5).
Point C as the Gingival distal margin	• X coordinate Victory series bracket = *X* of target −1.23 mm(Horizontal offset Bracket A) • Y coordinate of Victory series bracket = *Y* of target +1.08mm(Vertical offset Victory series bracket) (Fig. 6).

### Curve determination of the orthodontic bracket

1. When Points A, B and C are known from the determination of landmark point step, the curves of the orthodontic bracket fitting surface can be determined.
2. Point A as the inferior mesial margin and Point B as the gingival mesial margin will form the “Mesial curve” (Fig. 3).

**Fig. 3:**
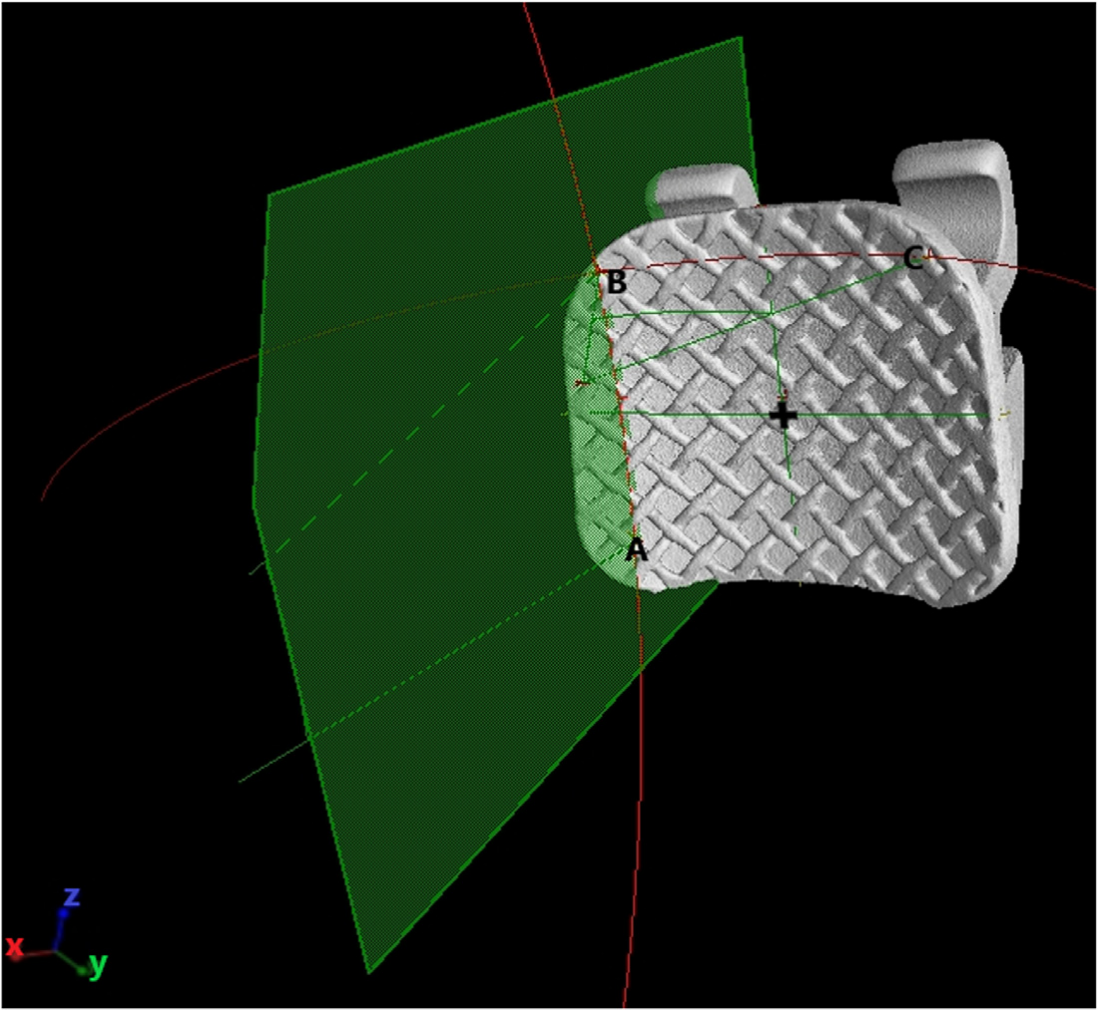
Point A as the inferior mesial margin and Point B as the gingival mesial margin will form the “Mesial curve”.
3. Point B as the gingival mesial margin and Point C as the gingival distal margin will form the “Gingival curve” (Fig. 4).

**Fig. 4:**
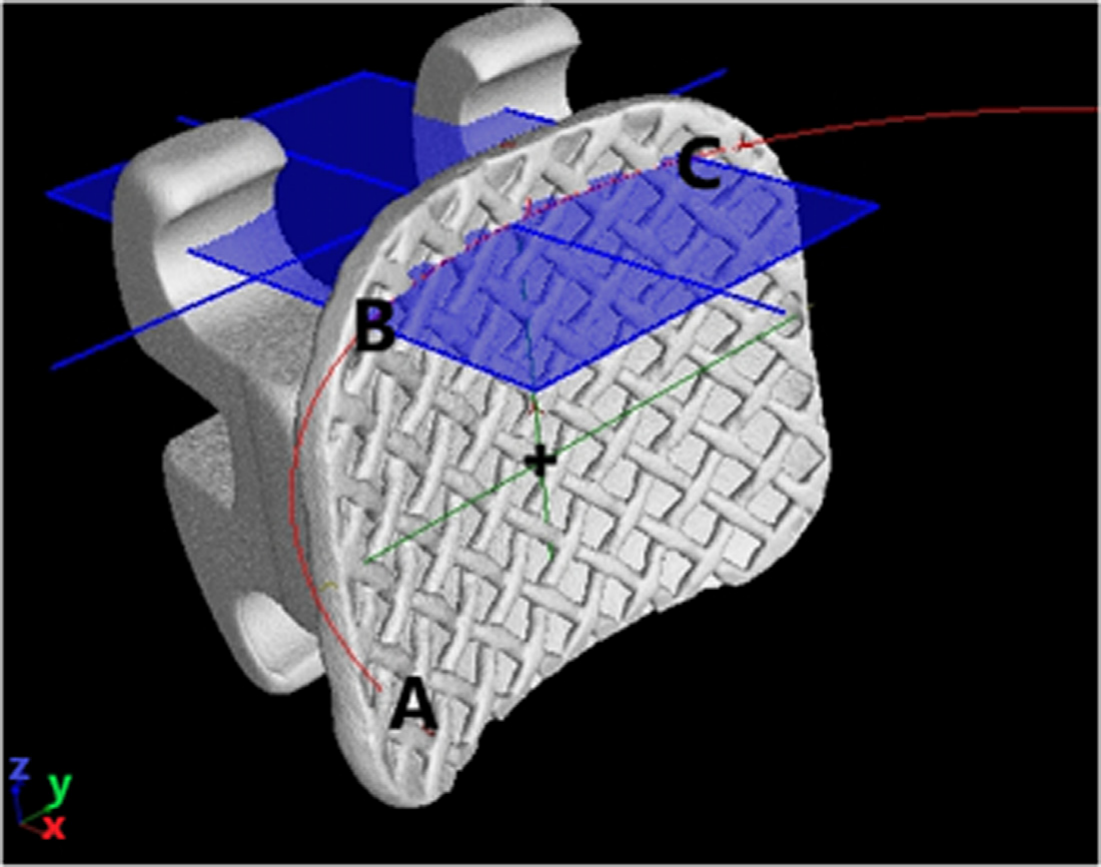
Point B as the gingival mesial margin and Point C as the gingival distal margin will form the “Gingival curve”.

### Orthodontic bracket curvature

In order to assess the curvature of the orthodontic bracket and how it relates to the contact surface on the tooth surface the curvature must be assessed with the closest fitting circle, followed by the determination of the angle. The angle is formed between two radii from the origin point of the best fitting circle and the landmark points of Points A and B respectively and between two radii from the origin point of the best fitting circle and the landmark points of Points B and C respectively.

*Determine the curvature of the orthodontic bracket surface:*

**Fig. 5:**
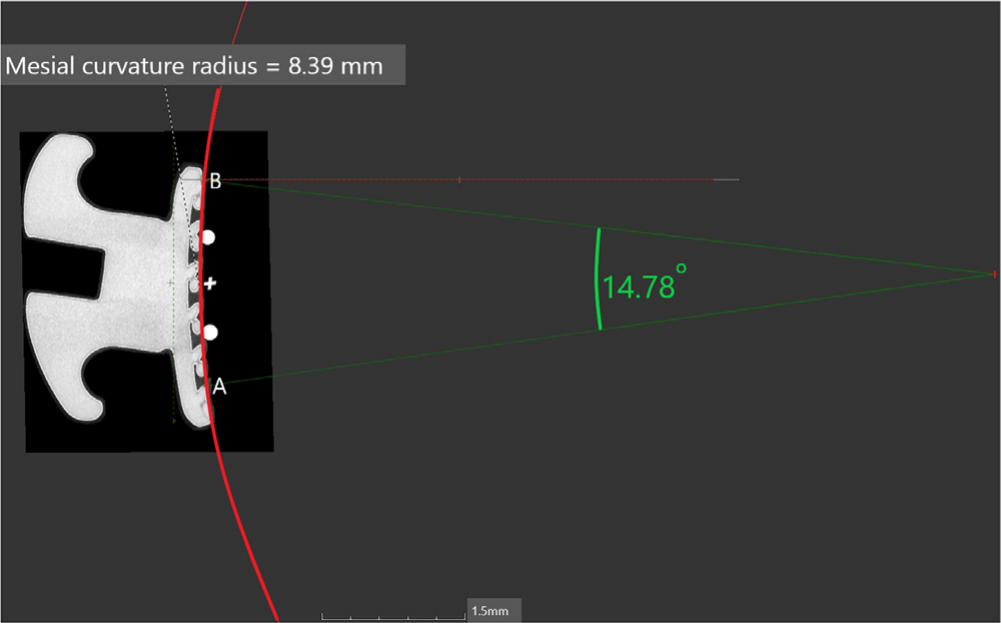
The mesial curve.

1. The Mesial curve will run between Point B and Point A (Fig. 3) and the gingival curve will run from Point B to Point C (Fig. 4).
2. The length of the curve measured for both the mesial and gingival curve using the measuring tool on VG Studio max 3.2.5 (Heidelberg, Germany 2018) and the midpoint of each curve is determined i.e. Midpoint of Curve (represented by “+” on Figs. 5, 6) is the centre point of the best fitting circle.

**Fig. 6:**
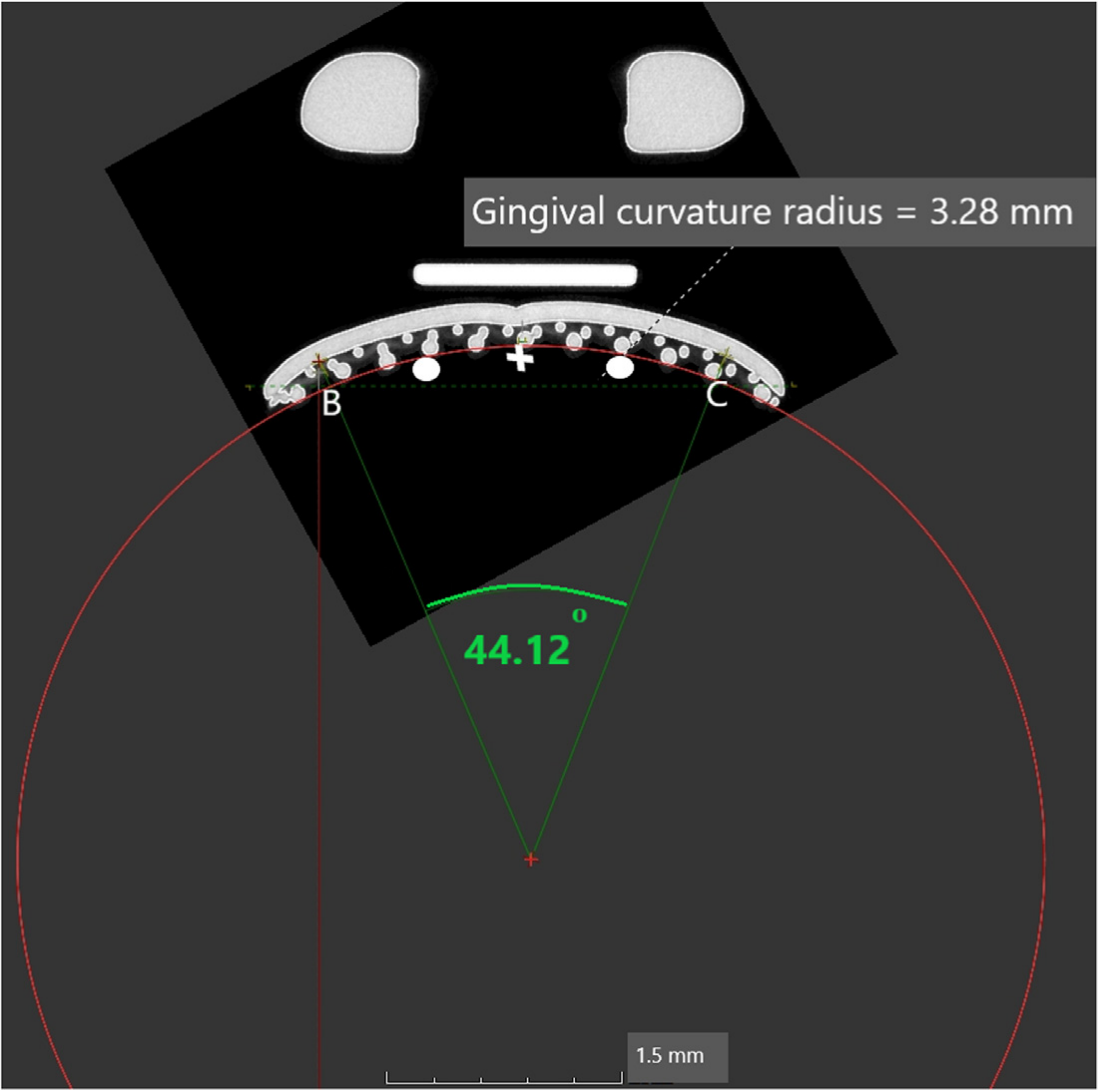
The gingival curve.
3. For the Mesial curve, an additional point is placed midway between the “Midpoint of the curve” and Point A and midway between the Midpoint of the curve and Point B (Fig. 5).
4. For the gingival curve, an additional point is placed midway between the “Midpoint of the curve” and Point B and midway between the Midpoint of the curve and Point C (Fig. 6).
5. A total of 5 Points are now present on each curve to allow for standardization in fitting of the best fitting circle.
6. The best fitting circle is fitted to the curve which passes through these 5 points on the curve using the circle tool in VG Studio max 3.2.5 (Heidelberg, Germany 2018) (Figs. 5, 6).
7. The origin of the circle is marked and two radii are constructed using line segments starting at the origin and terminating at Point A and Point B for the mesial curve and Point B and Point C for the gingival curve (Figs. 5, 6).
8. The angle between radii of the mesial curve is measured in degrees (Fig. 5).
9. The angle between the radii of the gingival curve is measured in degrees (Fig. 6).
6. The angular and radii measurements are then recorded on an Excel spreadsheet for the mesial curve and the gingival curve.

## Measurement parameters of teeth

### Height–width determination of teeth

1. Once the tooth digitized, the width of the tooth was measured from contact point to contact point.
2. The measured width and cusp tip is used to determine the central axis of the tooth (Fig. 7).

**Fig. 7:**
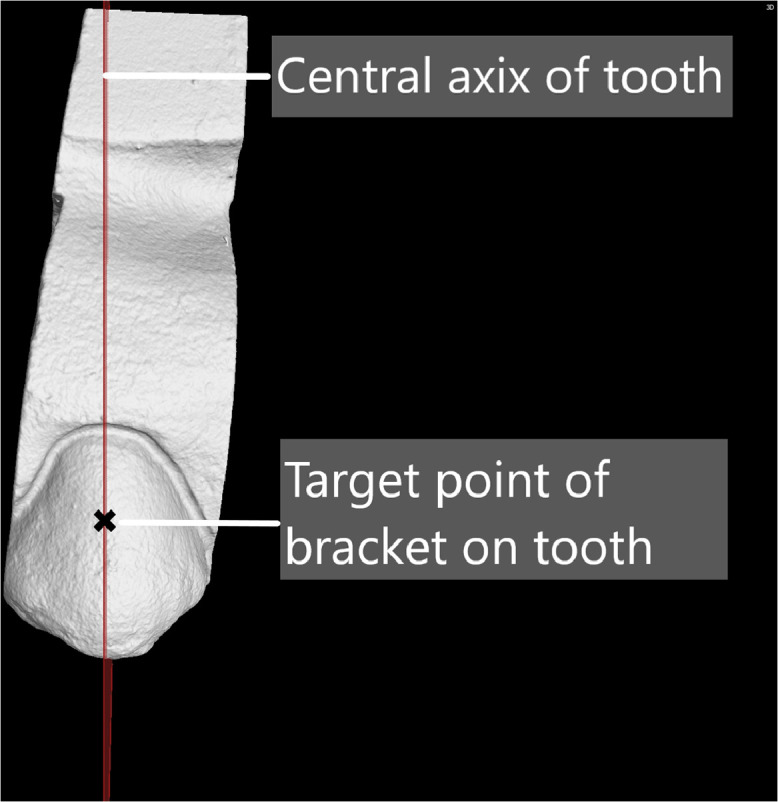
Central axis of the tooth.
3. The assessed orthodontic brackets have a prescription of 4 mm and therefore the “target point was placed on the central axis line 4 mm from the cusp tip (Fig. 7).
4. The x, y and z coordinates of the target point on the tooth is recorded.

### Orthodontic bracket placement on teeth

The co-ordinate points were determined for the orthodontic brackets in relation to the 4 mm orthodontic bracket prescription and represented in Table 3 for the first tooth as per Fig. 7. These co-ordinate points were determined using the target point on the tooth and the vertical and horizontal offsets from the bracket (Table 2). The co-ordinate points are plotted on the tooth in order to create a mesial and gingival curvature.

**Table 3:** Co-ordinates on a tooth for each bracket.

Tooth number 1	x	y	z
Target area on tooth located at	0,63	−5,7	−2,87
Victory series point A	1,86	−5,33	−3,95
Victory series point B	1,86	−5,11	−1,79
Victory series point C	−0,60	−5,68	−1,79
Innovation point A	1,93	−5,27	−4,09
Innovation point B	1,93	−5,14	−1,65
Innovation point C	−0,67	−5,61	−1,65
Bioquick point A	1,92	−5,29	−4,02
Bioquick point B	1,92	−5,06	−1,72
Bioquick point C	−0,66	−5,63	−1,72

The mesial curve is determined from Point A to Point B and the gingival curve is determined from Point B to Point C.

### Curve determination of the teeth

The images of the teeth are sliced at these landmark points, exposing their curvatures. The teeth are sliced from Point A to Point B in the vertical plane and from Point B to Point C in the horizontal plane.

1. Each curve is isolated and investigated separately i.e. Mesial curve: Point A to Point B, Gingival curve: Point B to Point C.
2. The length of the mesial curve measured using the measuring tool on VG Studio max 3.2.5 (Heidelberg, Germany 2018) and a landmark point is placed at the centre of the curve i.e. Midpoint of the curve.
3. An additional landmark point is place midway between Point A and the Midpoint of the curve.
4. An additional point landmark is place midway between Point B and the Midpoint of the curve (Fig. 8).

**Fig. 8:**
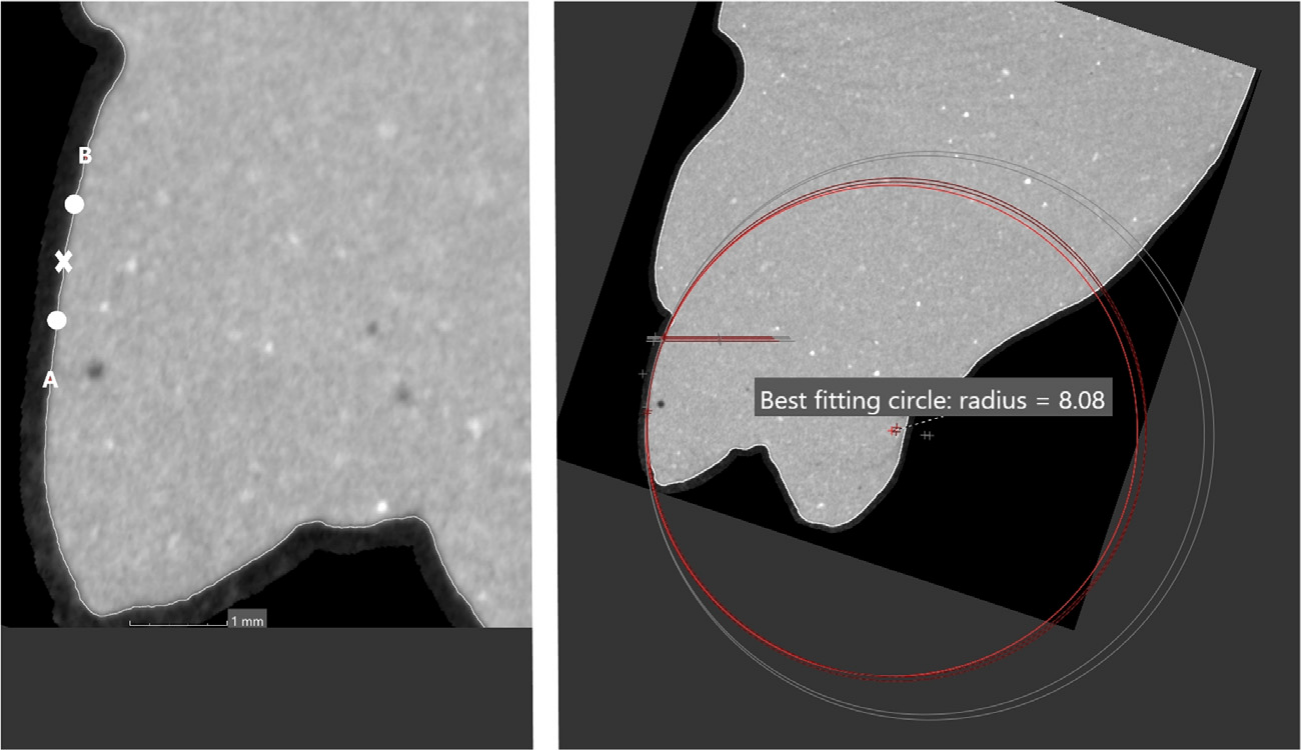
Additional landmark midway between Point B and the Midpoint of the curve.
5. A total of 5 landmark points are now present on the curve being investigated.
6. A circle is then fitted over these points using the circle tool within the VG Studio max 3.2.5 (Heidelberg, Germany 2018) program, which most closely fits the arc. The origin of the circle is automatically placed by the VG Studio max 3.2.5 (Heidelberg, Germany 2018). A radius is extended from the origin of the circle to Point A and another radius is extended from the origin to Point B. The angle between the radii was recorded as the central angle (Fig. 8).
7. The same process is repeated for the gingival curve providing the following points placed on it:
  • The curve extends from Point B to Point C
  • The arc length was measured and landmark point is placed at the midpoint of the curve.
  • An additional landmark point was place midway between Point B and the midpoint of the curve.
  • An additional point was place midway between Point C and the midpoint of the curve.
8. A circle is then fitted over these 5 landmark points using the circle tool within the VG Studio max 3.2.5 (Heidelberg, Germany 2018) program, which most closely fitted the arc. The origin of the circle is automatically placed by the VG Studio max 3.2.5 (Heidelberg, Germany 2018). A radius is extended from the origin of the circle to point B and another radius is extended from the origin to point C. The angle between the radii of point B and point C is then recorded (Fig. 9).

**Fig. 9:**
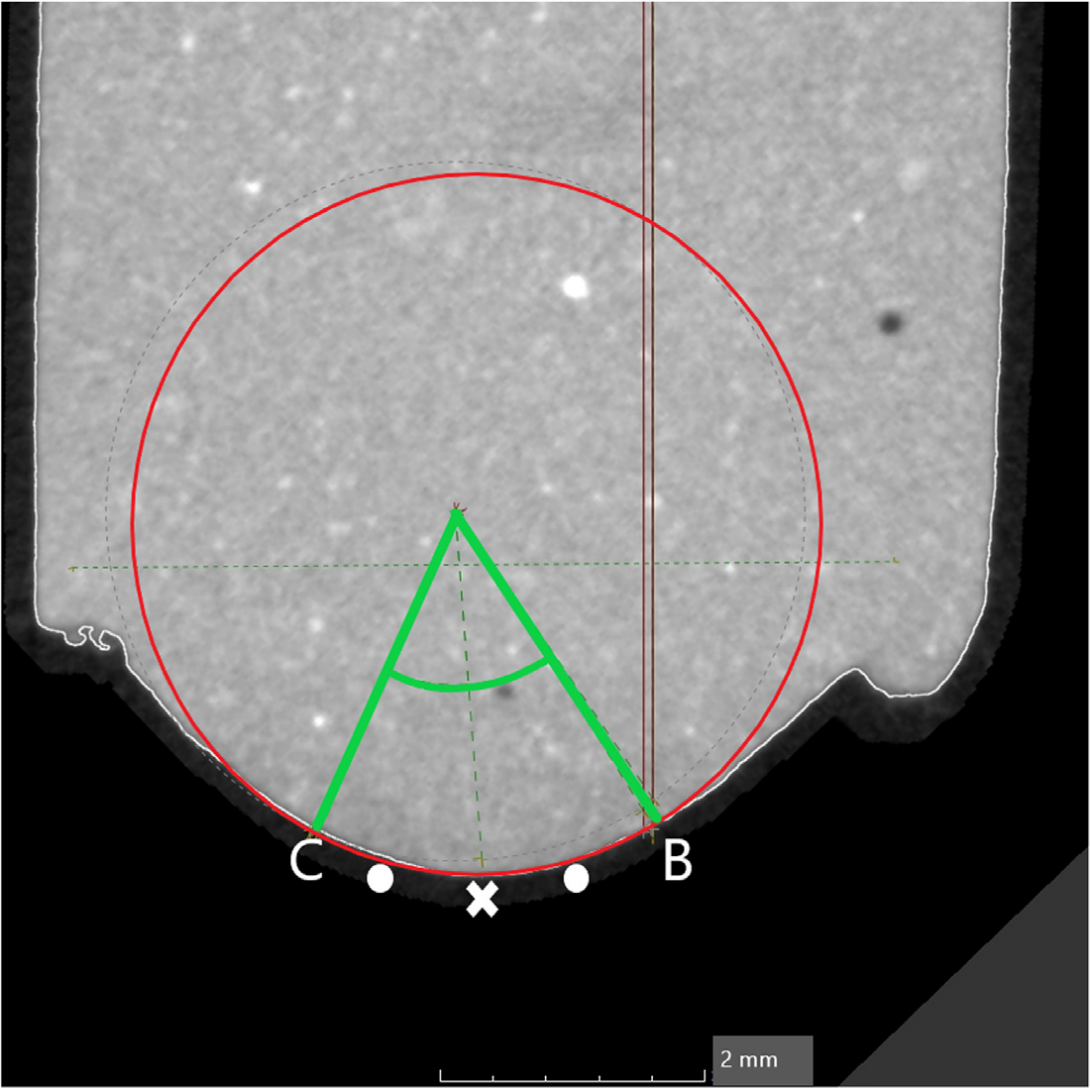
The angle between the radii of point B and point C.
9. Orthodontic bracket coordinates on each tooth result in a:
  • mesial and gingival angle measurement for the Victory series (3 M Unitek) bracket.
  • mesial angle and gingival angle measurement for the Innovation (GAC) bracket.
  • mesial and gingival angle measurement for the Bioquick (Forestadent) bracket.
10. These values are transferred to excel spreadsheet. The angles of the three brackets are then compared to the angles of the curvatures on the tooth of each brand to determine which bracket has a curvature which is closest to the teeth.

## Declaration of Competing Interest

The authors confirm that there are no conflicts of interest.
